# Rare variant analysis of whole genome sequenced juvenile idiopathic arthritis multiplex pedigrees identifies rare variants in *NOD2* and *ACVR1*

**DOI:** 10.1093/g3journal/jkag193

**Published:** 2026-07-17

**Authors:** Cecile N Avery, Edgar J Hernandez, Sandy G Kazuko, John F Bohnsack, Marc Sudman, Sampath Prahalad, Kaitlin Radmall, Aimee O Hersh, Anthea Letsou, Lynn B Jorde

**Affiliations:** Department of Biomedical Informatics, Vanderbilt University Medical Center, Nashville, TN 37203, United States; Department of Human Genetics, University of Utah, Salt Lake City, UT 84112, United States; Department of Human Genetics, University of Utah, Salt Lake City, UT 84112, United States; Department of Pediatrics, University of Utah, Salt Lake City, UT 84112, United States; Division of Rheumatology, University of Cincinnati College of Medicine and Cincinnati Children's Hospital Medical Center, Cincinnati, OH 45229, United States; Department of Pediatrics, Emory University School of Medicine, Atlanta, GA 30322, United States; Department of Human Genetics, University of Utah, Salt Lake City, UT 84112, United States; Department of Pediatrics, University of Utah, Salt Lake City, UT 84112, United States; Department of Human Genetics, University of Utah, Salt Lake City, UT 84112, United States; Department of Human Genetics, University of Utah, Salt Lake City, UT 84112, United States

**Keywords:** whole-genome sequencing, autoimmunity, pediatric, rare variant, *ACVR1*, *NOD2*, rare disease

## Abstract

Juvenile idiopathic arthritis is a complex rheumatic disease that is influenced by environmental and genetic factors. Linkage and genome-wide association studies have identified genes that contribute to the risk of developing juvenile idiopathic arthritis but are limited in their ability to identify disease-risk variants of large effect. Penetrant, heritable risk variants can be detected in high-risk families, but such cases are uncommon due to the low prevalence of juvenile idiopathic arthritis. This study utilizes whole-genome sequencing of 23 multiplex families, the largest such cohort to date, to discover variants and genes relevant to JIA pathogenesis. Pathogenic variants in *NOD2* associated with Blau syndrome, an ultra-rare Mendelian inflammatory disorder, are the most recurrent variants in the cohort, consistent with previous reports that milder presentations of Blau syndrome are oftentimes misdiagnosed as juvenile idiopathic arthritis. For the first time, however, rare variants in *ACVR1* and *SMAD6*, integral components of the Bone Morphogenic Protein pathway, are found to be associated with juvenile idiopathic arthritis. Identified *ACVR1 v*ariants map to critical protein domains. AlphaFold modeling predicts that the *ACVR1* interaction with its inhibitor OGT is disrupted by these variants, indicating that the patient-mutated protein has a gain-of-function phenotype. *Drosophila melanogaster* expressing either a wild-type or patient-mutated version of *ACVR1* exhibit embryonic lethality, with the mutant exhibiting 1.4-fold greater lethality than wild-type. The combination of family-based cohorts for gene discovery, AI-based computational tools, and animal model studies for tests of variant function underscores shared disease pathogenesis between JIA and monogenic disorders of immunity and connective tissue.

## Introduction

Juvenile idiopathic arthritis (JIA) is a rheumatic disease with an estimated prevalence of one in 1,000 and results in progressive joint damage and disability if left untreated (Prahalad et al. 2010). This condition predominantly manifests in children between the ages of 2 and 4 years old but can be diagnosed at any age under 16 (Prakken et al. 2011). JIA is a complex trait characterized by substantial phenotypic heterogeneity and is clinically divided into three primary subtypes: systemic, oligoarticular (four or fewer affected joints), and polyarticular (affecting more than four joints). Among these, systemic JIA is the rarest and most severe and appears to be genetically distinct from the other subtypes (Ombrello et al. 2017). Nevertheless, even within the oligoarticular and polyarticular JIA subtypes, genetic heterogeneity presents a challenge in pinpointing disease-associated genetic variation.

Family-based studies represent a powerful approach to understand the genetic basis for disease (Funauchi et al. 2000; Prahalad et al. 2000) and have been effective in establishing a genetic component in JIA risk. Studies of monozygotic twins with JIA estimate a concordance rate of approximately 20% to 40% (Glass and Giannini 1999; Funauchi et al. 2000; Prahalad et al. 2000), but paired dizygotic twin concordance estimates are not available. Pedigree-based studies using the Utah Population Database have shown that the relative risk (RR) of JIA among siblings of an affected proband is 11.6 (95% CI = 4.9 to 27.5) (Prahalad et al. 2010), indicative of familial risk factors. For comparison, rheumatoid arthritis (RA) has a sibling RR of 4.6 (95% CI = 3.0 to 7.2) (Hemminki et al. 2009). The elevated sibling RR for JIA, relative to RA, aligns with the notion that early onset forms of genetic diseases tend to exhibit a greater degree of genetic causation, making them excellent targets for genetic analysis (Jasperson et al. 2010; Naj and Schellenberg 2017).

To date, a limited number of genes have been associated with increased risk of JIA. Genome-wide association studies (GWAS) and fine-mapping studies have continued to replicate HLA associations first reported in linkage analyses (Thompson et al. 2004) and have identified 22 gene associations, most of which underscore the relevance of T cells in JIA pathophysiology (López-Isac et al. 2021). Because of their case-control design, GWAS are well powered to identify relatively common disease-causing variants but can fail to identify rare (AF < 0.01) pathogenic variants.

Whole-genome sequencing of multiplex families, in which a pathogenic variant can appear in multiple affected persons, offers the potential to detect rare disease-causing variants. Due in part to its low prevalence, such studies are uncommon for JIA but have revealed novel monogenic risk loci that eluded detection by other methods. For instance, homozygous alleles in *LACC1* across five consanguineous families with systemic JIA (Wakil et al. 2015), and in one consanguineous family with three cases of rheumatoid factor negative polyarticular JIA (Rabionet et al. 2019), show clear relevance to immune system dysfunction through pathological inflammasome activation not detected by GWAS. Furthermore, a hemizygous variant in *FOXP3,* shared among four siblings, has been proposed as the causal variant for recurrent treatment-refractory juvenile psoriatic arthritis (Alzyoud et al. 2021), a subtype of JIA that is accompanied by a scaly rash on the skin and nails. Phenotypic overlap with Mendelian diseases, such as Blau Syndrome, has also been reported in cases with a family history of JIA (Furness et al. 2022). Exome and genome sequencing studies of JIA suggest that rare variants tend to lie in regulatory regions active in immune cells (Wong et al. 2017) and modulating immunological pathways (Farh et al. 2014). While we have gleaned many useful insights from large-scale GWAS studies, there is a distinct lack of sequencing studies of multiplex JIA families.

We present whole-genome sequencing of the largest cohort of multiplex families to date, enabling identification of pathogenic variants relevant to JIA predisposition. Using a variant prioritization algorithm for rare mendelian diseases, we identified candidate genes with compelling evidence for relevance to JIA biology and explored their impact in computational and animal models. Identification of disease-associated genes and pathogenic variants serves as a foundation for comprehensive functional analyses, contributing to conceptual advances in our understanding of JIA and leveraged to improve patient outcomes across the autoimmune arthritis spectrum.

## Material and methods

### Ascertainment of samples

Retrospective identification of candidate JIA families for sequencing included samples from biobanks at the University of Utah and Cincinnati Children's Hospital Medical Center. Samples selected for sequencing were prioritized on number of affected siblings, concordance of JIA subtype between siblings, and younger age of onset. Both parents were also sequenced, except in instances when DNA was not available. Two pedigrees lack sequencing data for both parents (pedigrees 1,145 and 110,105), two lack paternal sequencing (pedigrees 11,103 and 30,532), and one lacks maternal sequencing data (pedigree 689). In total, 23 multiplex pedigrees were sequenced with case counts ranging from two to four per pedigree (Supplementary Table 1). Three pedigrees lacked paternal genotyping, and two pedigrees wholly lacked parental genotyping. For those two pedigrees, DNA from an unaffected sibling was isolated and sequenced. This cohort, including parents, is mostly of genetic European ancestry (*n* = 82), with some samples of Hispanic or Latino ancestry (*n* = 17), totaling 99 individuals. Thirty-three affected siblings were diagnosed with oligoarticular JIA, 20 with polyarticular JIA, five with polyarticular JIA (rheumatoid factor positive), and one case lacks information on JIA subtype. Phenotypes of affected parents were recorded during sample collection and study enrollment. Affected parent phenotypes in this cohort are limited to JIA (*n* = 4), RA (*n* = 3), or general autoimmunity (*n* = 2).

### Patient and public involvement

Patients or the public were not involved in the design, or conduct, or reporting, or dissemination plans of our research.

### Whole-genome sequencing and variant calling

Blood-derived DNA was used for sequencing. Six samples were whole-genome sequenced with unique molecular identifiers for PCR-free library preparation, while 93 were sequenced with PCR-based preparation. Each of the six PCR-free samples represents one case from six different pedigrees and were sequenced as a pilot. Paired-end whole genome sequencing (2 × 150 bp) was performed on an Illumina Novaseq 6000 to a target coverage of 30×. Additional information on sample processing is described in the Supplementary Materials.

### Rare variant prioritization

Rare variant prioritization was performed with GEM (v7.0), an AI-based genomic interpretation and clinical decision support tool that expedites rare variant review by incorporating biological and clinical data to decrease the number of candidate variants being considered (De La Vega et al. 2021). Metrics such as inheritance pattern, allele frequency in gnomAD v2.1.1 (Collins et al. 2020), ClinVar 1.7 (Landrum et al. 2018), and known disease associations or phenotypes were incorporated into a forward-chain probability algorithm to predict if a variant is disease-causing. 1000 Genomes Project (Sudmant et al. 2015) (GRCh37) genomes were used as controls (CEU (CEPH from Utah, Northern European ancestry)/GBR (British from England and Scotland) = 85, FIN (Finnish) = 30 AMR (Admixed American) = 25). Sample-level outputs contain candidate single nucleotide polymorphisms (SNPs) ordered by a GEM score, expressed as a Bayes factor that represents the likelihood that the variant contributes to disease divided by the likelihood that it does not. GEM is typically used as a diagnostic support tool; scores >1 are notable and scores above 2 are considered likely diagnostic. Given our use of GEM as a discovery tool, a filter for a minimum GEM score of 0.7 was applied, which is considered “substantial evidence” in standard practice (Kass and Raftery 1995). Details for this analysis are included in the Supplementary Materials.

### External singleton and trio cohorts

A whole-genome sequenced cohort of polyarticular and oligoarticular JIA singletons (*n* = 115) was collected from previously published datasets (BioProject accession: PRJNA343545 [Wong et al. 2017], PRJNA1054595 [Avery et al. 2024]), as well as a collection of 23 early onset RA trios. These data were processed with the same pipeline as the multiplex family cohort. Demographic and phenotypic information on these samples is limited to sex and JIA subtype.

### Protein interaction prediction (ACVR1 and OGT [O-GlcNAc Transferase])

Computational modeling of the impact of missense variants on ACVR1 variants observed in pedigrees was performed with AlphaFold3. Protein sequences used as input for AlphaFold3 (Abramson et al. 2024) were obtained on Uniprot: Q04771 · ACVR1_HUMAN, O15294 · and Q7KJA9 · Q7KJA9_DROME (Sxc). For each variant of interest, the appropriate amino acid position was edited. AlphaFold was used to generate several predicted structures, binding interactions, and confidence metrics. Further, refined binding predictions were produced by analyzing the predicted protein structures in ChimeraX (Meng et al. 2023). Predicted Aligned Error (PAE) plots were generated for each combination of ACVR1 and OGT interactions. PAE is defined as the expected positional error at residue X, measured in Ångströms (Å), if the predicted and actual structures were aligned on residue Y. Therefore, PAE is a measure of confidence that the domains are well packed and that the relative placement of the domains in the predicted structure is correct. This produces a “grid system” of the proteins’ predicted folding, and potential interaction domains. PAE plots were color-coded to reflect local confidence, or pLDDT. The PAE plot visualizes the confidence of the protein structures and domains that are predicted to interact, and the combined structure.

### Fly crosses

Drosophila were selected to model the impact of R160G on ACVR1. Drosophila lines containing UAS-linked *ACVR1* variant R160G (*UAS*-*ACVR1^R160G^*) and WT *ACVR1* (*UAS*-*ACVR1^+^*) (Supplementary Table 2) were generated with the PhiC31 integrase-mediated transgenesis system targeting chromosome III on a stock strain (#8622) at BestGene Inc. Cuticular phenotypes were assessed in flies expressing the wild-type and mutant transgenes ubiquitously using the *Ubi-Gal4* driver. Additional experimental details can be found in Supplementary File S1.

## Results

Our JIA cohort consists of 59 cases with oligoarticular and polyarticular disease with a sex ratio of 2.9:1 female to males. The median age of onset within the cohort, when available, is approximately 2.7 years with a range of 0.8 to 14.4 years old. Twelve pedigrees have an affected parent with JIA, RA, or other autoimmune disorder (Table 1). In these pedigrees, disease risk transmission was presumed to be autosomal dominant. In all other families, transmission was assumed to be autosomal recessive. However, these assumptions are not rigid and do not limit discovery. GEM considers multiple inheritance patterns including *de novo* mutations. Candidate variants that score highly on other metrics of pathogenicity will still be reported, but the final GEM score will be moderately impacted by a mismatch between assumed inheritance pattern and the observed zygosity of the variant (De La Vega et al. 2021).

**Table 1.** Cohort summary.

	Total count
Male	14
Female	40
Median (known) age of onset^a^	2.7 years old
Pedigrees with an affected parent(s)	12
Pedigrees with unaffected parents	11
Oligoarticular subtype	28
Polyarticular subtype	25

^a^Data missing for some individuals.

### Inheritance patterns and variant prioritization

GEM analysis was performed on all 23 multiplex families in the cohort as sets of parent–child pairs or trios of both parents and child, depending on the availability of genotype data. In one instance, three affected siblings lacked parental genotype data (pedigree 1,145). These cases were run individually as singletons, and results were compared to a sequenced, unaffected sibling. After removing incidental findings, such as risk variants within *BRCA1* or *CFTR*, and filtering results on phenotypic relevance (Phevor [Singleton et al. 2014] score >0.9), 57 candidate variants remained (Supplementary Table 3). By requiring that affected siblings share the same variant, the number of candidates is reduced by 51.9% to 26 variants. Ten of 23 sequenced families (43%) have a plausible candidate across all affected individuals in a pedigree (Table 2). All the candidate variants identified in these families are found in a heterozygous state. The assumption of autosomal dominant inheritance matched the zygosity observed by these candidate variants apart from pedigree 1,069, which features three affected children and two unaffected parents. Four pedigrees with presumed autosomal dominant transmission did not result in a plausible risk variant.

**Table 2.** Families with concordant GEM results.

Pedigree	Gene	Variant coordinates (GRCh37)	GEM score	Total cases	Parents genotyped
1,017	*NOD2*	16:50744822:C:T	4.74 to 4.77	3	Yes
1,130	*NOD2*	16:50744823:G:A	4.68 to4.72	4	Yes
207	*NOD2*	16:50744822:C:T	3.08 to3.09	3	Yes
1,022	*NOD2*	16:50745581:C:T	0.962 to1.04	3	Yes
45,139	*ACVR1*	2:158634708:G:C	0.82 to1.63	4	Yes
30,532	*ACVR1*	2:158634825:G:A	0.75 to 0.82	2	Mother
11,103	*SMAD6*	15:66996283:C:CT	1.07 to 1.88	4	Mother
1,069	*AKT1*	14:105239351:G:A	0.74 to 0.79	3	Yes
1,019	*PKLR*	1:155261636:C:T	1.86 to 1.88	2	Yes
17,599	*EIF2AK2*	2:37349802:G:A	0.83 to 0.86	2	No

### Identification of known and novel candidate genes

*NOD2* appeared multiple times as a plausible candidate gene across unrelated families. Eight siblings across four families were heterozygous for known (pedigrees 1,017, 1,130, 207) or likely (pedigree 1,022) pathogenic variants within *NOD2*. Pedigree 1,130 demonstrates an extensive maternal family history of JIA, including a pair of identical twin sisters (only one was sequenced), a son, affected mother, and three additional extended maternal relatives. Pedigrees 1,022 and 1,017 each feature two affected siblings with an affected mother, while pedigree 207 contains two affected siblings and an affected father (Supplementary Fig. 1). The GEM scores for the known *NOD2* candidate variants range from 3.08 to 4.77, well above the typical diagnostic threshold of 2, and represent the top 11.7% of GEM scores observed in the cohort. These variants are reported in ClinVar, which highly influences GEM scores as strong evidence aiding diagnosis. These variants are associated with Blau Syndrome, an ultra-rare, NOD2-associated mendelian autoinflammatory disorder with phenotypic similarity to JIA (ie arthritis, uveitis, and cutaneous rashes present in most cases). The *NOD2* variant segregating in pedigree 1,022 has a lower GEM score (0.962 to 1.04), likely because this variant is reported as a variant of uncertain significance (VUS).

**Fig. 1.**
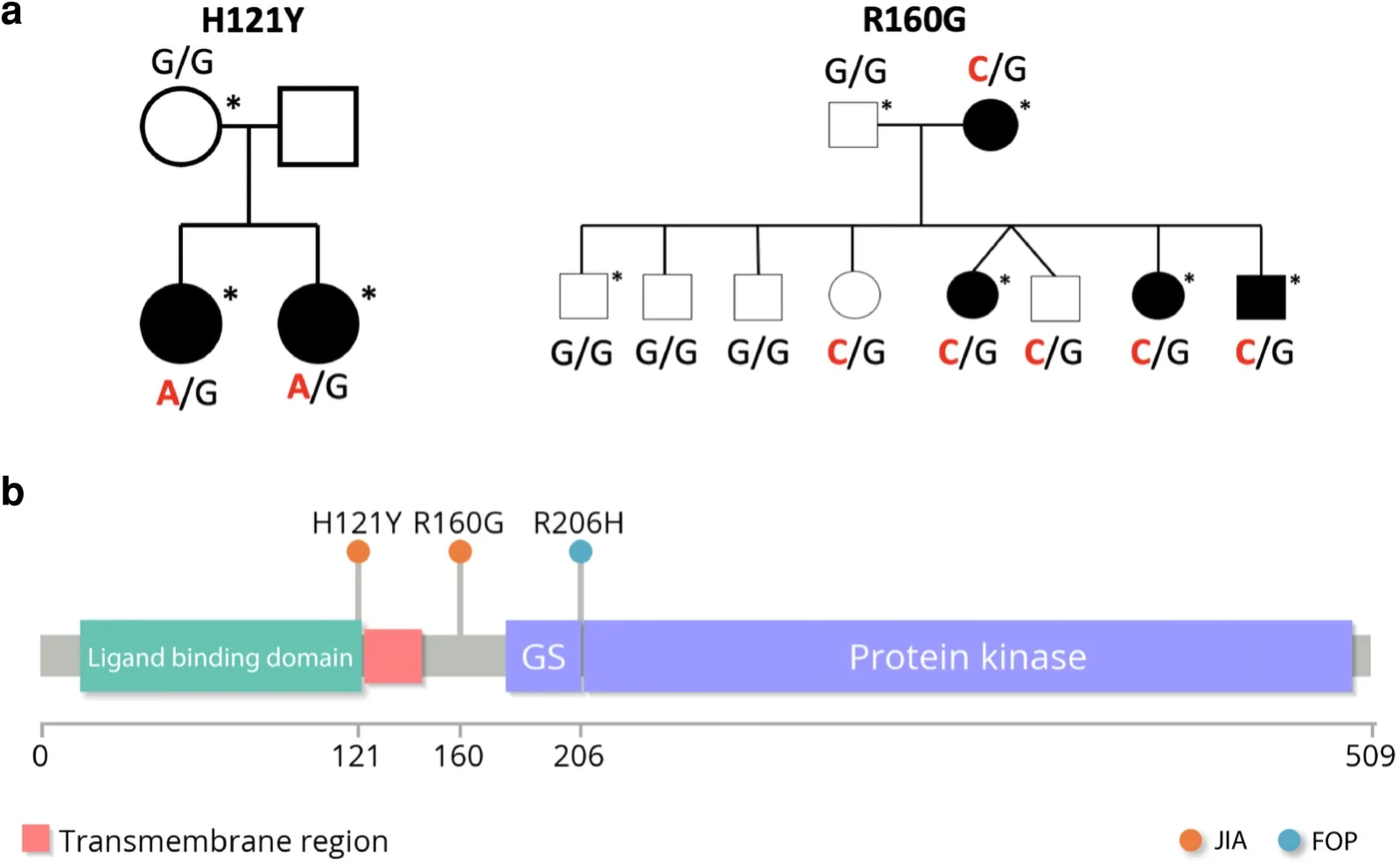
Multiplex pedigrees and ACVR1 variant distribution. a) Two multiplex pedigrees harbor rare variants in ACVR1. Sanger sequencing was performed on unaffected siblings and revealed incomplete penetrance. b) Lollipop diagram of ACVR1 indicating the relative positions of the two ACVR1 variants identified in JIA pedigrees and the most common causal variant for FOP. GS, Glycine serine-rich domain. *Whole-genome sequenced.

Two predicted pathogenic variants within exons of *ACVR1* were observed in two families (pedigrees 30,532 and 45,139) as heterozygotes (Fig. 1a). The GEM scores for the reported *ACVR1* variants range from 0.75 to 1.63 and are reported as VUS in ClinVar. *ACVR1* H121Y has been submitted three times without a specified phenotype/condition, and a single submission indicating congenital heart disease. *ACVR1* R160G has only one submission as a VUS without an accompanying phenotype. The *ACVR1* variants detected in our multiplex pedigrees (R160G, H121Y) are predicted to fall near the annotated boundary of the glycine–serine-rich (GS) domain and within the transmembrane domain, respectively (Fig. 1b). In pedigree 45,139, R160G is transmitted by the mother, affected with adult RA, to three children with oligoarticular JIA in an autosomal dominant inheritance pattern. Sanger sequencing of additional, ungenotyped children revealed that five of the eight children inherited *ACVR1^R160G^* (Supplementary Fig. 2) (Fig. 1a). The two unaffected children may still develop JIA in the future as at the time of sample ascertainment, they were under the age of 16; however, discordance of JIA has been described in monozygotic twin pairs, suggesting incomplete penetrance (Glass and Giannini 1999; Funauchi et al. 2000; Prahalad et al. 2000). In pedigree 30,532, *ACVR1^H121Y^* was not transmitted from the mother, who does not have autoimmune arthritis but is affected with scleroderma; genotype data are not available for the father.

In pedigree 1,069, which contained three affected offspring and unaffected parents, a maternally inherited variant in *AKT1*, which regulates cell proliferation and survival (Duggal et al. 2018), passed our filtering criteria and was present in all three offspring. This suggests autosomal dominant inheritance with incomplete penetrance in the mother, but we cannot rule out other plausible alternative explanations, such as additional pathogenic structural variants not detected in these analyses, methylation, and environmental exposures.

### Enrichment analysis of candidate genes

Gene set enrichment analysis (GSEA) was performed over the set of all unique candidate genes (*n* = 34) with a GEM score >0.7 and Phevor scores > 0.9 in the pedigree data set. Gene sets with a statistically significant (adj. *P* < 0.05) overlap were predominantly associated with biological processes such as macromolecule biosynthesis, negative regulation of cell death, and phosphorylation. Candidate genes that predominantly contribute to the overlaps across these gene sets included *AKT1*, *NOD2*, *EIF2AK2*, *ACVR1*, and *SMAD6*. Notably, *NOD2*, *ACVR1*, and *SMAD6* are among the most frequently implicated genes between the discovery cohort and replication cohort.

### External extension of results

A cohort of whole-genome sequenced singletons was assembled from previously published datasets as well as 23 trios of early-onset RA (<30 years old), yielding a dataset of 115 whole-genome sequenced singletons. This cohort contained a case diagnosed with interstitial lung disease and seropositive JIA that was later diagnosed as COPA syndrome (autoimmune interstitial lung, joint, and kidney disease) after identifying a known pathogenic variant in *COPA* (E241K) (Watkin et al. 2015). This variant was appropriately prioritized by GEM with a score of 3.32 (Supplementary Table 4). Of note, interstitial lung disease develops in a subset of adult RA cases (Kim et al. 2009), and a novel *COPA* variant farther downstream was recently proposed as causal in a case of RF + polyarticular JIA (Bader-Meunier et al. 2021). Despite the frequency of *NOD2* variants in the multiplex family cohort, only one candidate *NOD2* indel (16:50745722:AGGCCAGGCAACTCACCAAT:A, GEM score = 0.69) was identified in the singleton cohort. Additional predicted pathogenic variants in *ACVR1* were not detected. However, a missense heterozygous variant in *SMAD6* at 15:67073388:T:C was observed (GEM score = 0.79), approximately 105 amino acids downstream from the 15:66996283:C:CT frameshift variant observed in pedigree 11,103 present in three affected siblings and the affected mother (Table 1).

Several candidate variants with high GEM scores >2 (eg SAMHD1, ENPP1) unique to the singleton cohort were detected, and only two genes harbored multiple, unique pathogenic variant candidates. Two individuals were heterozygous for variants in *RELA* (11:65429656:G:A, 11:65422356:A:AGGGGCC), which encodes the p65 subunit of the transcription factor NF-κB, a critical regulator of the immune system and inflammatory processes (Vallabhapurapu and Karin 2009). Variants in RELA leading to RELA haploinsufficiency are associated with autoinflammatory disease (An et al. 2023); these variants may contribute to the dysregulation of the NFKB pathway and increase risk of JIA. In addition, two unrelated individuals share a rare, predicted pathogenic indel in *MME* (3:154834478:AC:A, gnomAD AF = 0.0002).

### Computational prediction of ACVR1 pathophysiology

Rare variant analysis revealed *ACVR1* as a candidate gene with plausible biological relevance to the pathophysiology of JIA, contributing to JIA risk within pedigrees. Activating mutations in *ACVR1* have been previously implicated in another genetic disorder of connective tissue, fibrodysplasia ossificans progressiva (FOP) (Kaplan et al. 2008). Expression of ACVR1 in *Drosophila* has been used to document hyperactive Dpp (the Drosophila Bone Morphogenic Protein [BMP] ortholog) signaling resulting from expression of ACVR1^R206H^ [FOP] (Le and Wharton 2012). Thus, to directly assess the functionality of ACVR1^R160G^, transmitted from an affected mother to three cases, we turned to *Drosophila*, the organism in which the OGT/ACVR1 interaction was first documented (Moulton et al. 2020) and more broadly, a powerful model to assess functionality of human disease variants.

The positions of the newly identified JIA-associated *ACVR1^R160G^* and *ACVR1^H121Y^* mutations map closely to *ACVR1^R206H^* (the most frequent pathogenic variant observed in FOP) (Le and Wharton 2012), and around the ACVR1 GS domain, where negative regulation by O-GlcNAcylation is proposed to occur. We used AlphaFold3, an artificial intelligence program that produces accurate protein structure and interaction predictions (Abramson et al. 2024), to map the interaction of ACVR1 with its inhibitor O-GlcNAc transferase (OGT; Super Sex Combs [Sxc] in Drosophila). As expected, we found that the Sxc catalytic domain interacts with the ACVR1 GS domain (Fig. 3a). This interaction is disrupted by *ACVR1^R206H^* [FOP] (Fig. 3b), providing new mechanistic insight into this gain-of-function disease allele. As was the case for *ACVR1^R206H^* [FOP], the Sxc/ACVR1 interaction is severely disrupted by the JIA-associated allele *ACVR1^H121Y^*. The *ACVR1^R160G^* allele also disrupts the Sxc/ACVR1 interaction, albeit less dramatically than either *ACVR1^R206H^* [FOP] or *ACVR1^H121Y^* (Fig. 3, c and d). These computational data support pathogenicity for the *ACVR1^R160G^* and *ACVR1^H121Y^* missense alleles in JIA and provide a foundation for functional studies in the Drosophila model.

### ACVR1^R160G^ expression in *Drosophila* hyperactivates BMP signaling

**Fig. 2.**
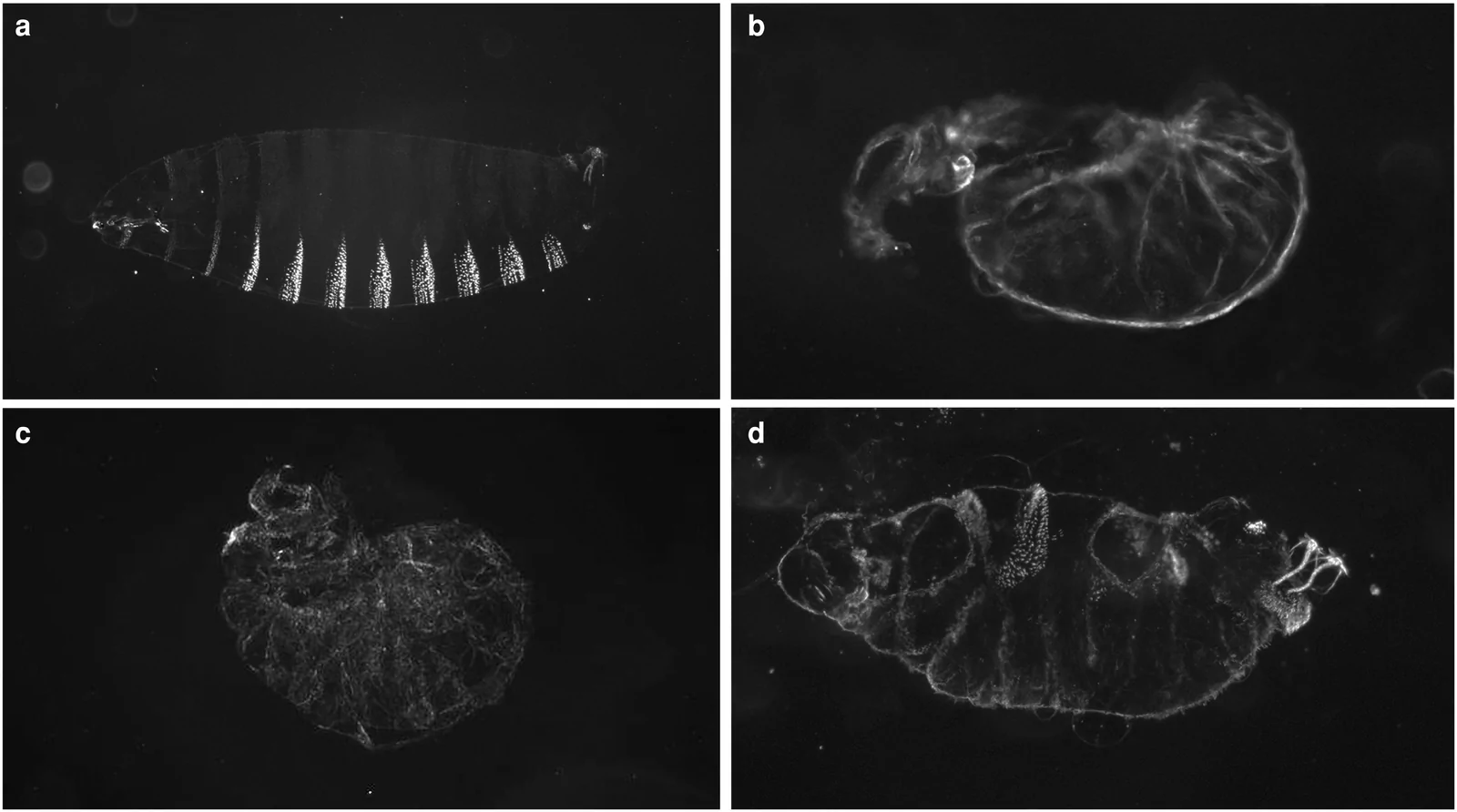
ACVR1^R160G^ expression results in aberrant cuticle formation in *Drosophila* embryos. a) Normal morphology of a wild-type cuticle compared to lines expressing (b) *UAS-Sxc^+^*. c) *UAS-ACVR1^+^*, d) *UAS-ACVR1^R160G^.*

While the severity of OGT/ACVR1 disruption was greatest for ACVR1^R206H^ [FOP] and ACVR1^H121Y^, the disruption was more subtle for the OGT/ACVR1^R160G^ pairing. The *Drosophila* ortholog of *ACVR1* is *saxophone* (*sax*). In *Drosophila* embryos, inappropriate activation of *sax* elevates Dpp/BMP signaling and leads to an incompletely penetrant embryonic lethality (∼30%) that is associated with signature cuticle defects in dorsal closure (Moulton et al. 2020). We found that ectopic expression of either the *UAS-ACVR1^+^* or *UAS-ACVR1^R160G^* transgene in *Drosophila* embryos via ubiquitously expressed *Ubi-Gal4* (*Ubi > ACVR1^+^* or *Ubi > ACVR1^R160G^*) results in an analogous incompletely penetrant embryonic lethality, with cuticles derived from ACVR1 transgenic flies exhibiting the same hyperactive Dpp/BMP signaling cuticle phenotype that we documented previously for *sax* (Fig. 2). Moreover, lethality measured in *Ubi > ACVR1^R160G^* animals (37%, *n* = 400) is significantly greater than that in *Ubi > ACVR1*^+^ animals (26%, *n* = 472) (Χ2(1, *N* = 872) = 12.1 *P* < 0.001) (Supplementary Table 5). These data demonstrate that both wild-type ACVR1 and the disease-associated variant function in *Drosophila*, with the variant form producing even higher levels of Dpp signaling than the wild-type. These observations are consistent with the prediction that ACVR1^R160G^ is a dominant gain-of-function variant that could contribute to the pathophysiology of JIA.

**Fig. 3.**
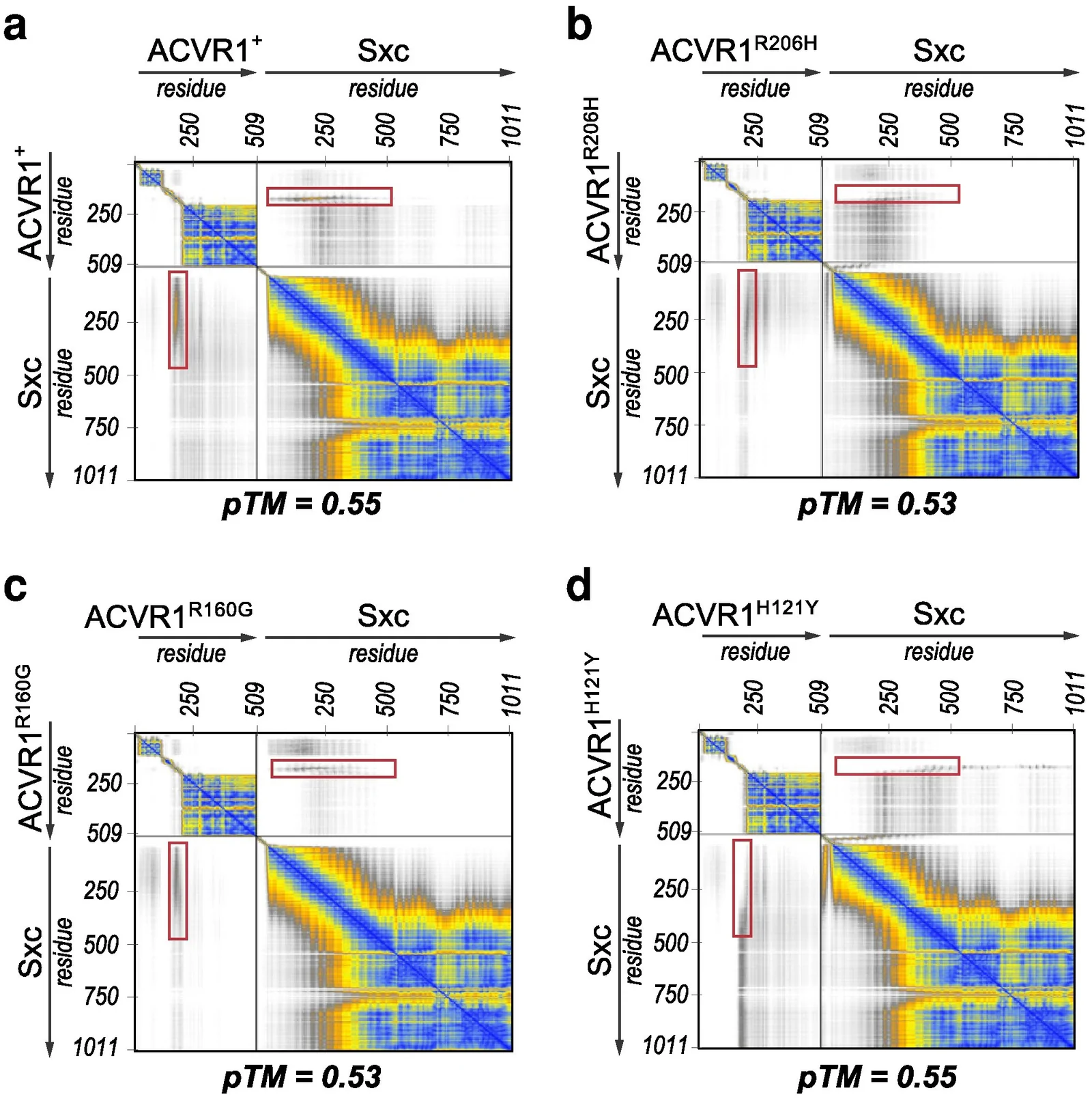
Alphafold3 PAE ACVR1-Sxc Binding Plots. PAE plots visualizing model confidence of the structure and binding of ACVR1 variants and Sxc by predicted local distance difference test (pLDDT), which is scaled from 0 to 100. Higher pLDDT correspond to higher confidence and a more accurate prediction. Predicted template modeling (pTM) scores are reported for each predicted interaction; scores above 0.5 indicate that the predicted interaction is similar to the hypothetical true structure. Encircled by the red boxes, are the binding interactions between Sxc and ACVR1's GS domain. In ACVR1^+^, binding is shown by a strong grey-yellow band (a). The FOP variant, ACVR1^R206H^, is located just outside the GS domain and within the protein kinase domain, shows a predicted loss of binding within the GS domain, and different interactions in the regions upstream (b). In contrast, a milder variant R160G, which is located between all major motifs, has decreased binding overall (c). The H121Y variant is located between the ligand binding domain and the transmembrane region of ACVR1. A complete loss of binding within the GS region and expanded interaction beyond is predicted (d). pLDDT: Dark blue = pLDDT > 90, light blue = 90 > pLDDT > 70, yellow = 70 > pLDDT > 50, orange = pLDDT < 50.

## Discussion

The phenotypic heterogeneity of JIA and its relative rarity present a significant challenge in identifying novel, rare genetic risk variants. Very few studies have definitively described monogenic forms of JIA, and in fact, several JIA cases that have undergone genetic sequencing are reported to have been re-diagnosed with a Mendelian condition upon considering new genetic information (Imundo et al. 2011; Torgutalp et al. 2019; Chen et al. 2022; Furness et al. 2022). We utilized a cohort of multiplex families to identify heritable genetic risk and observe its transmission to more than one child. By targeting this cohort of familial JIA cases, we reduce the overall genetic heterogeneity of the cohort and simultaneously improve our detection of penetrant, inherited risk variants.

Within our cohort, four families carry rare variants in *NOD2*, the most commonly represented candidate gene in this study. NOD2 plays a critical role in immune cell function and NF-κB signaling and has been associated with Crohn's disease (Ogura et al. 2001) and Blau syndrome (Miceli-Richard et al. 2001). Several of the JIA pedigrees transmitting pathogenic variants in *NOD2* have a positive family history of autoimmunity and appear to exhibit autosomal dominant transmission. Despite the prevalence of *NOD2* variants in our pedigrees, only one rare variant in *NOD2* was found in the external cohort of singletons. This observation could result from selectively ascertaining JIA cases with a positive family history of autoimmunity or JIA and multiple affected siblings, which produces the intended effect of detecting heritable, highly penetrant pathogenic variants. Overall, rare, pathogenic variants in the genes highlighted in this study, although only found in a minority of familial JIA cases, are relevant to JIA pathology broadly.

Blau syndrome, an autosomal dominant disease that commonly manifests as polyarticular arthritis in children 2 to 4 years old, has an estimated prevalence of only one in 1,000,000 (considerably rarer than JIA). Additional symptoms such as dermatitis and uveitis are common (Miceli-Richard et al. 2001). While studies of Blau syndrome are limited, clinical manifestations of this disease can vary between and within families and can result in a misdiagnosis of JIA. A previous study reported that exome sequencing of a family with a strong family history of JIA revealed pathogenic variant R334Q in *NOD2* in all affected family members (Furness et al. 2022). Our study reports an additional four families with Blau syndrome, previously ascertained as JIA. R334W and R334Q, frequently reported Blau syndrome variants (Matsuda et al. 2020), were observed in two families and a single family, respectively. A third reported variant, R587C, is also reported in Blau syndrome cases (Matsuda et al. 2020) and seen in one multiplex family in our cohort. In addition, a heterozygous frameshift variant (N637Sfs*120) resulting in a 19-base pair deletion was detected in the replication cohort. This transcript is predicted to undergo nonsense-mediated decay, but constraint metrics from gnomAD v2.11 suggest that NOD2 is haplosufficient (Karczewski et al. 2020). Therefore, this variant is less likely to contribute to this individual's disease risk, but worthy of experimental follow-up given the relevance of variants in *NOD2*. We propose that substantial phenotypic overlap between less severe manifestations of Blau syndrome and JIA, resulting in occasional misdiagnosis, should be investigated as an avenue for understanding the pathophysiology of autoimmune arthritis broadly. In addition, families with a history of JIA may benefit from being considered for Blau syndrome.

*ACVR1*, which appeared as a probable candidate gene in two families, has been previously associated with FOP (Shore et al. 2006). FOP is a rare autosomal dominant disorder, and associated mutations appear to be concentrated near the GS and kinase domains of the protein. The variants observed in our JIA cases are upstream of pathogenic variants associated with FOP and predicted to impact interactions in the GS domain. JIA-associated variants are likely autosomal dominant, given: (i) the transmission pattern observed in the families, (ii) the phenotype of our *Drosophila* model of ACVR1^R160G^ and computational predictions observed in ACVR1^R160G^ and ACVR1^H121Y^, and (iii) known FOP variants are also autosomal dominant. The most common FOP mutation is an amino acid substitution, R206H, within the GS domain (Shore et al. 2006), resulting in the activation of BMP signaling in the absence of ligand (Fukuda et al. 2009) and/or overactivation of BMP signaling in response to ligand (Billings et al. 2008). FOP patients experience episodic inflammatory soft tissue flare-ups that result in cumulative heterotopic ossification and disability (Kaplan et al. 2008). While JIA is considerably less severe than FOP, JIA patients appear to be susceptible to similar mechanisms of heterotopic ossification observed in response to intraarticular corticosteroid injections and the inflammatory environment surrounding the affected joints (Ringold et al. 2011). In the context of our animal model, the activity of the variant ACVR1^R160G^ (which inappropriately elevates BMP signaling) is functionally distinct from wild-type and is itself either inappropriately activated or fails to be appropriately suppressed. The rarity of ACVR1^R160G^ has contributed to the paucity of evidence for its pathogenicity. We contribute familial and experimental data to support reclassification from a VUS in the context of autoimmune arthritis, although additional follow-up is still needed.

ACVR1 is a BMP signaling molecule that, upon activation, forms a tetrameric complex with type-2 receptors and phosphorylates SMAD proteins (Valer et al. 2019). SMAD6 inhibits BMP signal transduction (Imamura et al. 1997), and chondrocytes of *SMAD6 −/−* mice exhibit elevated BMP signaling, resulting in aberrant differentiation at the growth plate (Estrada et al. 2011). Within our cohort, we observed a frameshift (R231Sfs*72) in SMAD6 shared by three affected children and an affected parent with a positive family history of autoimmune disease. In silico predictions of this late frameshift by MutationTaster (Steinhaus et al. 2021) suggest that the truncated protein (−37 amino acids) would result in a loss of SMAD-1 binding and subsequent loss of inhibition of BMP-SMAD1 signaling (Imamura et al. 1997; Estrada et al. 2011). Given that known pathogenic variants in *ACVR1* aberrantly activate BMP signaling, we propose that these candidate variants in *ACVR1* and *SMAD6* share the biological mechanism of sensitizing BMP activation. Experimental follow-up is needed to confirm the consequence of this frameshift on the transcript and/or protein. BMPs, a member of the larger TGF-β family, have wide-ranging effects on a variety of tissues (Wang et al. 2014) and future work could determine whether these rare variants primarily drive disease processes of the immune system, skeletal system, or both.

## Conclusion

Whole-genome sequencing of multiplex families affected by JIA provides a unique opportunity to investigate inherited disease risk. This study represents the largest whole-genome sequencing effort using multiplex families to date, yielding insights into the genetic landscape of JIA. The implications of a strong family history of JIA and another autoimmune disease should prompt consideration of genetic testing when alternative diagnoses are suspected, such as Blau syndrome. This diagnostic strategy is supported by the findings of this study as well as previous reports. Of particular interest are the rare variants we found in *ACVR1* which were shared among multiple affected siblings and an affected parent. Heterotrophic ossification observed in FOP, associated with pathogenic variants in ACVR1, and occasionally noted in JIA suggest a shared, complex disease etiology. Further functional analyses of the variants described here are warranted and can serve as a catalyst for future efforts to identify and sequence high-risk JIA families. Such endeavors are poised to significantly enhance our understanding of pediatric autoimmune arthritis and, ultimately, improve patient care.

## Supplementary Material

jkag193_Supplementary_Data

## Data Availability

This study was reviewed and approved by the University of Utah Institutional Review Board (IRB) under IRB#:00009542. Patients who enrolled in the JIA biobank, which compose much of the study cohort, were not explicitly consented to publish their sequencing data publicly. All sequencing data generated as part of this study are store securely at the University of Utah. This data can be made available upon reasonable request. Details are located in the Supplementary data access file (Supplementary File 2). *Drosophila* lines are available upon request. Supplemental material available at G3 online.
