# Supplementary material for: Rare variant analysis of whole genome sequenced juvenile idiopathic arthritis multiplex pedigrees identifies rare variants in NOD2 and ACVR1
Source: G3 (Bethesda). 2026 Jul 17;16(9):jkag193. doi: 10.1093/g3journal/jkag193 (PMC13535375; doi:10.1093/g3journal/jkag193)
Supplement: Supplementary file 1 [file jkag193_supplementary_data.zip › File_S1_G3-2026-406889.docx]

Supplementary Material (File S1)

**Methods**

**Whole genome sequencing and variant calling**

An average median coverage of 48x was achieved across the cohort. All samples passed quality control metrics of genotype quality and heterozygosity. Additionally, predicted versus reported familial relatedness and sex were assessed with Peddy^1^ to detect non-paternity, sample swaps, and consanguinity. Genomes were aligned to build GRCh38 with BWA-MEM^2^. Indels and single nucleotide variants (SNVs) were called with Sentieon^3^ and annotated with VEP (v104)^4^. Genome coordinates were transferred from GRCh38 to build GrCh37 to utilize GEM^5^, a rare-variant prioritization tool.

**Rare variant prioritization**

GEM provides a probabilistic score, reported as a Bayes factor, per gene to guide interpretation of the ranked output, and determines when all the most likely pathogenic variants have been reviewed. An annotated VCF, all relevant human phenotype ontology (HPO) terms per individual, and pedigree information were used as input for GEM. Because GEM is limited to use in trios, duos, and singletons, all multiplex families were split into several trios or duos. If parental genotypes were missing, each affected sibling was run as a singleton. GEM’s performance is not significantly impacted when a case is run as an individual versus a trio on a variant-by-variant basis^5^ . However, gene prioritization improves with parental genotypes. GEM also computes ancestry of the proband and relatedness to parents when provided so that sample errors, non-paternity, or consanguinity can be flagged.

Genes identified by GEM were filtered individually based on phenotypic relevance, reported as a Phevor^6^ score, of >.9. These genes were subsequently filtered on a GEM score of >.7, which has been shown to recover 95% of all true positives. Within each pedigree, the remaining SNVs and indels were filtered to include only those variants shared among siblings. Candidate indels were reviewed on Integrative Genomics Viewer (IGV)^7^, and in silico predictions for the impact on the protein product were generated with MutationTaster^8^.

Molecular Signatures Database (MSigDB) was used to perform gene set enrichment analysis^9^ on the unique genes remaining after filtering and review. Gene sets were selected from the following human collections curated within MSigDB: Positional gene sets (C1), immunologic signature gene sets (C7), cell type signature gene sets (C8), canonical pathways (CP) derived from KEGG^10^, Reactome^11^, and WikiPathways^12^ databases, hallmark gene sets (H), and Gene Ontology (GO) gene sets.

**Cloning of Wild-Type and the Patient ACVR1 R160G variant**

A putative causal variant, R160G, was introduced via PCR site-directed mutagenesis (SDM) (Table S2) into a donor plasmid containing *ACVR1* (Addgene plasmid #23822). Wild-type (WT) and mutant *ACVR1* inserts were ligated into a pBID-UAS (Addgene plasmid #35198) backbone via a T4 ligase reaction (NEB) and transformed into NEB5-α cells.

**Fly Stocks**

Insertion of WT ACVR1 and the patient variant ACVR1 was confirmed with PCR, and the remaining integrase sites were removed. *Ubi-Gal4* (#32551), *Ap-Gal4* (#3041), and *A9-Gal4* (#8761) were obtained from the Bloomington *Drosophila* Stock Center (BDSC). Flies were maintained on cornmeal/molasses/agar medium at room temperature (22C) unless otherwise stated.

**Fly Phenotyping**

Lethality was assayed at the embryonic, larval, and pupal stages of development in the progeny of Ubi-Gal4/CyO virgin females mated to either UAS-AVCR1*^R160G^* or UAS-AVCR^+^ males, with100 eggs collected per line per experiment. Lethality experiments were performed four times. All crosses were maintained at 25C.  Cuticle phenotypes of progeny exhibiting embryonic lethality were viewed after mounting samples in One-Step Mounting Medium (30% CMCP-10, 13% lactic acid, 57% glacial acetic acid).

1.	Pedersen, B. S. & Quinlan, A. R. Who’s Who? Detecting and Resolving Sample Anomalies in Human DNA Sequencing Studies with Peddy. *Am J Hum Genet* **100**, 406–413 (2017).

2.	Li, H. Aligning sequence reads, clone sequences and assembly contigs with BWA-MEM. (2013).

3.	Weber, J. A., Aldana, R., Gallagher, B. D. & Edwards, J. S. Sentieon DNA pipeline for variant detection - Software-only solution, over 20× faster than GATK 3.3 with identical results. (2016) doi:10.7287/PEERJ.PREPRINTS.1672V2.

4.	McLaren, W. *et al.* The Ensembl Variant Effect Predictor. *Genome Biol* **17**, 122 (2016).

5.	De La Vega, F. M. *et al.* Artificial intelligence enables comprehensive genome interpretation and nomination of candidate diagnoses for rare genetic diseases. *Genome Med* **13**, 1–19 (2021).

6.	Singleton, M. V. *et al.* Phevor combines multiple biomedical ontologies for accurate identification of disease-causing alleles in single individuals and small nuclear families. *Am J Hum Genet* **94**, 599–610 (2014).

7.	Robinson, J. T., Thorvaldsdóttir, H., Wenger, A. M., Zehir, A. & Mesirov, J. P. Variant review with the integrative genomics viewer. *Cancer Res* **77**, e31–e34 (2017).

8.	Steinhaus, R. *et al.* MutationTaster2021. *Nucleic Acids Res* **49**, W446–W451 (2021).

9.	Subramanian, A. *et al.* Gene set enrichment analysis: A knowledge-based approach for interpreting genome-wide expression profiles. *Proc Natl Acad Sci U S A* **102**, 15545–15550 (2005).

10.	Ogata, H. *et al.* KEGG: Kyoto Encyclopedia of Genes and Genomes. *Nucleic Acids Res* **27**, 29–34 (1999).

11.	Fabregat, A. *et al.* Reactome pathway analysis: a high-performance in-memory approach. *BMC Bioinformatics* **18**, (2017).

12.	Pico, A. R. *et al.* WikiPathways: Pathway Editing for the People. *PLoS Biol* **6**, e184 (2008).
