# Supplementary material for: Rare variant analysis of whole genome sequenced juvenile idiopathic arthritis multiplex pedigrees identifies rare variants in NOD2 and ACVR1
Source: G3 (Bethesda). 2026 Jul 17;16(9):jkag193. doi: 10.1093/g3journal/jkag193 (PMC13535375; doi:10.1093/g3journal/jkag193)
Supplement: Supplementary file 1 [file jkag193_supplementary_data.zip › File_S2_G3-2026-406889.docx]

**DATA ACCESS POLICY**

(File S2) Requests for access to the data should be made in writing to the corresponding author and must include: the names of the individuals and of their respective institutional affiliations who will be provided access to the data; the aims of the proposed research that will be conducted using the data; the procedures the requesting party will employ to ensure the privacy and confidentiality of the data; and a description of what information will be made publicly available in any materials or publications generated from the proposed research. The proposed study must have reasonable and well-justified scientific goals.

**Parties responsible for evaluating requests**

Corresponding author Dr. Cecile Avery, senior author and project supervisor Dr. Lynn Jorde, and the Chair of the University of Utah Institutional Review Board (IRB).

**Criteria and procedure used to evaluate requests**

The corresponding author will forward the request to the other granting parties, who will each evaluate the request based on the scientific merit of the proposed aims and the adequacy of the proposed procedures for protecting participant privacy and confidentiality. Each party may ask the requesting investigator for further clarification before providing a recommendation. Expected response time to request for data access is approximately two months.

**Conditions for gaining access to the data**

- The requesting party must execute a material transfer agreement (MTA) provided by the granting parties.
- Confidentiality of the data provided must be guaranteed, and all liability resulting thereof, by the requesting party in writing, as part of the material transfer agreement.
- The investigator cannot share the data with any other individual or party not named in the approved request.
- Any results or materials intended for public release or publication that were generated using the data must be shared with the corresponding author for review by the granting parties prior to submission. The granting parties will have 45 days for this review, and the requesting party will accommodate all reasonable changes consistent with the conditions outlined herein.
- Results of the proposed research will not be used nor licensed for commercial purposes.

**Usage or types of requests that would lead to denial of access**

- The proposed research involves the investigation of phenotypes other than juvenile idiopathic arthritis.
- The proposed study design could potentially identify individual study participants.
- The proposed research includes or anticipates the publication of individual genotypes or sequence data.
- The requesting party is unable to provide adequate guarantees for the privacy and confidentiality of the data.
