# Supplementary material for: Rare variant analysis of whole genome sequenced juvenile idiopathic arthritis multiplex pedigrees identifies rare variants in NOD2 and ACVR1
Source: G3 (Bethesda). 2026 Jul 17;16(9):jkag193. doi: 10.1093/g3journal/jkag193 (PMC13535375; doi:10.1093/g3journal/jkag193)
Supplement: Supplementary file 1 [file jkag193_supplementary_data.zip › Supplemental_Figures_G3-2026-406889.docx]

Figure S1. *NOD2* Pedigrees.

Shaded individuals had been diagnosed with juvenile idiopathic arthritis. Red shading indicates individuals diagnosed with an autoimmune condition. Asterisks indicate individuals selected for whole genome sequencing (DNA on additional members of the pedigree are available).

Figure S2. Sanger sequencing of pedigree 45139.

**Table S1.** Multiplex family sample table with phenotypes.

| **Kindred** | **Sample** | **Father** | **Mother** | **Sex** | **Affection** | **Phenotype** |
| --- | --- | --- | --- | --- | --- | --- |
| 49 | 2601 | 0 | 0 | 1 | 2 | Diabetes mellitus |
| 49 | 2600 | 0 | 0 | 2 | 2 | Autoimmunity |
| 49 | 119 | 2601 | 2600 | 1 | 2 | oJIA |
| 49 | 120 | 2601 | 2600 | 1 | 2 | pJIA |
| 49 | 121 | 2601 | 2600 | 1 | 2 | oJIA |
| 111 | 2595 | 0 | 0 | 1 | 2 | JIA |
| 111 | 2594 | 0 | 0 | 2 | 1 |  |
| 111 | 307 | 2595 | 2594 | 2 | 2 | JIA |
| 111 | 2596 | 2595 | 2594 | 2 | 2 | oJIA |
| 111 | 2597 | 2595 | 2594 | 2 | 2 | oJIA |
| 111 | 2598 | 2595 | 2594 | 2 | 2 | oJIA |
| 207 | 597 | 0 | 0 | 1 | 2 | JIA |
| 207 | 596 | 0 | 0 | 2 | 1 |  |
| 207 | 561 | 597 | 596 | 2 | 2 | pJIA |
| 207 | 562 | 597 | 596 | 1 | 2 | pJIA |
| 250 | 1657 | 0 | 0 | 2 | 1 |  |
| 250 | 1658 | 0 | 0 | 1 | 1 |  |
| 250 | 672 | 1658 | 1657 | 2 | 2 | pJIA (RF+) |
| 250 | 674 | 1658 | 1657 | 2 | 2 | pJIA |
| 250 | 673 | 1658 | 1657 | 2 | 2 | pJIA |
| 689* | 1630 | 0 | 0 | 1 | 1 |  |
| 689* | 1629 | 1630 | 0 | 2 | 2 | oJIA |
| 689* | 1631 | 1630 | 0 | 2 | 2 | oJIA |
| 689* | 1628 | 1630 | 0 | 2 | 2 | pJIA (RF+) |
| 1017 | 2166 | 0 | 0 | 1 | 1 |  |
| 1017 | 2165 | 0 | 0 | 2 | 2 | JIA |
| 1017 | 2167 | 2166 | 2165 | 2 | 2 | pJIA |
| 1017 | 2168 | 2166 | 2165 | 1 | 2 | pJIA |
| 1019 | 2291 | 0 | 0 | 1 | 1 |  |
| 1019 | 2290 | 0 | 0 | 2 | 2 | RA |
| 1019 | 2292 | 2291 | 2290 | 2 | 2 | pJIA |
| 1019 | 2293 | 2291 | 2290 | 1 | 2 | pJIA |
| 1022 | 2361 | 0 | 0 | 1 | 1 |  |
| 1022 | 2360 | 0 | 0 | 2 | 2 | Autoimmunity |
| 1022 | 2362 | 2361 | 2360 | 2 | 2 | pJIA |

Table S1. continued

| **Kindred** | **Sample** | **Father** | **Mother** | **Sex** | **Affection** | **Phenotype** |
| --- | --- | --- | --- | --- | --- | --- |
| 1022 | 2363 | 2361 | 2360 | 1 | 2 | pJIA |
| 1055 | 2113 | 0 | 0 | 2 | 1 |  |
| 1055 | 2114 | 0 | 0 | 1 | 1 |  |
| 1055 | 2004 | 2114 | 2113 | 2 | 2 | oJIA |
| 1055 | 2005 | 2114 | 2113 | 2 | 2 | pJIA |
| 1069 | 2490 | 0 | 0 | 1 | 1 |  |
| 1069 | 2489 | 0 | 0 | 2 | 1 |  |
| 1069 | 2491 | 2490 | 2489 | 1 | 2 | oJIA |
| 1069 | 2492 | 2490 | 2489 | 2 | 2 | oJIA |
| 1069 | 2493 | 2490 | 2489 | 1 | 2 | oJIA |
| 1130 | 2477 | 0 | 0 | 1 | 1 |  |
| 1130 | 2476 | 0 | 0 | 2 | 2 | pJIA |
| 1130 | 2480 | 2477 | 2476 | 1 | 2 | oJIA |
| 1130 | 2478 | 2477 | 2476 | 2 | 2 | oJIA |
| 1145 | 2568 | 0 | 0 | 2 | 1 |  |
| 1145 | 2566 | 0 | 0 | 1 | 2 | oJIA |
| 1145 | 2567 | 0 | 0 | 1 | 2 | oJIA |
| 1145 | 2569 | 0 | 0 | 1 | 2 | oJIA |
| 4194 | 8406 | 0 | 0 | 2 | 1 |  |
| 4194 | 8407 | 0 | 0 | 1 | 1 |  |
| 4194 | 8394 | 8407 | 8406 | 2 | 2 | pJIA |
| 4194 | 8395 | 8407 | 8406 | 2 | 2 | oJIA |
| 11103 | 57446 | 0 | 0 | 2 | 2 | JIA |
| 11103 | 57449 | 0 | 57446 | 2 | 2 | oJIA |
| 11103 | 57447 | 0 | 57446 | 1 | 2 | oJIA |
| 11103 | 57448 | 0 | 57446 | 1 | 2 | oJIA |
| 14064 | 107613 | 0 | 0 | 1 | 1 |  |
| 14064 | 107612 | 0 | 0 | 2 | 1 |  |
| 14064 | 107588 | 107613 | 107612 | 2 | 2 | oJIA |
| 14064 | 107587 | 107613 | 107612 | 2 | 2 | oJIA |
| 14064 | 107589 | 107613 | 107612 | 2 | 2 | oJIA |
| 14064 | 107611 | 107613 | 107612 | 1 | 2 | oJIA |
| 18256 | 74870 | 0 | 0 | 2 | 1 |  |
| 18256 | 74871 | 0 | 0 | 1 | 1 |  |
| 18256 | 74651 | 74871 | 74870 | 2 | 2 | oJIA |

Table S1. continued

| **Kindred** | **Sample** | **Father** | **Mother** | **Sex** | **Affection** | **Phenotype** |
| --- | --- | --- | --- | --- | --- | --- |
| 18256 | 75131 | 74871 | 74870 | 2 | 2 | oJIA |
| 24743 | 84457 | 0 | 0 | 2 | 1 |  |
| 24743 | 84458 | 0 | 0 | 1 | 1 |  |
| 24743 | 84456 | 84458 | 84457 | 2 | 2 | pJIA |
| 24743 | 85246 | 84458 | 84457 | 2 | 2 | pJIA |
| 29242 | 92654 | 0 | 0 | 2 | 1 |  |
| 29242 | 202206 | 0 | 0 | 1 | 1 |  |
| 29242 | 92652 | 202206 | 92654 | 2 | 2 | oJIA |
| 29242 | 127355 | 202206 | 92654 | 2 | 2 | pJIA |
| 30532 | 95069 | 0 | 0 | 2 | 2 | Autoimmunity |
| 30532 | 95068 | 0 | 95069 | 2 | 2 | pJIA |
| 30532 | 127142 | 0 | 95069 | 2 | 2 | pJIA |
| 45139 | 120512 | 0 | 0 | 1 | 1 |  |
| 45139 | 113811 | 0 | 0 | 2 | 2 | RA |
| 45139 | 123577 | 120512 | 113811 | 1 | 1 |  |
| 45139 | 113810 | 120512 | 113811 | 2 | 2 | oJIA |
| 45139 | 120509 | 120512 | 113811 | 2 | 2 | oJIA |
| 45139 | 120510 | 120512 | 113811 | 1 | 2 | oJIA |
| 52721 | _001 | 0 | 0 | 2 | 1 |  |
| 52721 | _002 | 0 | 0 | 1 | 1 |  |
| 52721 | 123679 | _002 | _001 | 2 | 2 | pJIA |
| 52721 | 123676 | _002 | _001 | 2 | 2 | pJIA |
| 52721 | _003 | _002 | _001 | 2 | 2 | pJIA |
| E_02 | SL88822 | 0 | 0 | 1 | 1 |  |
| E_02 | SL88821 | 0 | 0 | 2 | 1 |  |
| E_02 | SL88823 | SL88822 | SL88821 | 2 | 2 | pJIA (RF+), alopecia |
| E_02 | SL88824 | SL88822 | SL88821 | 2 | 2 | pJIA (RF+) |
| E_02 | SL414736 | SL88822 | SL88821 | 2 | 2 | pJIA (RF+) |
| 110105 | 1152 | 0 | 0 | 2 | 2 | oJIA |
| 110105 | 1154 | 0 | 0 | 2 | 2 | oJIA |

**Sex:** Male (1), Female (2)

**Affection:** Unaffected (1), Affected (2)

Father/Mother values indicate the sample ID of the parent. A value of 0 indicates that the sample ID does not exist.

*Affected mother not genotyped

Table S2. PCR primers.

| **ACVR1_45139_GT_F** | GCCAGCATGTGCTTGTTTAG |
| --- | --- |
| **ACVR1_45139_GT_R** | TGGAGGTTGGCCTCATTATTC |
| **ACVR1_45139_SDM_F** | CTCAATCCCGGAGACGTGGAGTATG |
| **ACVR1_45139_SDM_R** | GCGTTCTTGGTTGCGCCTTTTAAATTTTC |
| **F_kozak_xhoI_ACVR1** | GGGGCTCGAGCAAAATGGTAGATGGAGTG |
| **R_XhoI_stop_ACVR1** | CCCCCTCGAGTCAACAGTCAGTTTTC |
| **F_Sal_kozak_ACVR1*** | GGATGTCGACCAAAATGGTAGATGGAGTG |
| **R_Sal_stop_ACVR1*** | CCATGTCGACTCAACAGTCAGTTTTC |
| **UAS_ACVR1_start_F** | GTAACCAGCAACCAAGTAAATCAAC |
| **UAS_ACVR1_start_R** | CTTCGATAGTGCCATACTCCAC |
| **ACVR1_int2_F** | CACCAAGCGCTACATGGC |
| **ACVR1_int2_R** | CGAGTCAACAGTCAGTTTTC |
| **UAS_ACVR1_END_F** | CCTATGGAACTGATGAATGGGAG |
| **UAS_ACVR1_END_R** | CAATTATGTCACACCACAGAAG |

Table S5. Embryonic lethality

| **Line** | **Hatched** | **Not Hatched** | **Embryonic Lethality** |
| --- | --- | --- | --- |
| **ACVR1_WT** | 349 | 123 | 26.05% |
| **ACVR1_R160G** | 251 | 149 | 36.5% |
| **Total** | 600 | 272 | 31.19% |
